# Measuring crops in 3D: using geometry for plant phenotyping

**DOI:** 10.1186/s13007-019-0490-0

**Published:** 2019-09-03

**Authors:** Stefan Paulus

**Affiliations:** Institute of Sugar Beet Research, Holtenser Landstr. 77, 37079 Göttingen, Germany

**Keywords:** 3D plant scanning, Plant traits, Parameterization, Plant model, Point cloud

## Abstract

Using 3D sensing for plant phenotyping has risen within the last years. This review provides an overview on 3D traits for the demands of plant phenotyping considering different measuring techniques, derived traits and use-cases of biological applications. A comparison between a high resolution 3D measuring device and an established measuring tool, the leaf meter, is shown to categorize the possible measurement accuracy. Furthermore, different measuring techniques such as laser triangulation, structure from motion, time-of-flight, terrestrial laser scanning or structured light approaches enable the assessment of plant traits such as leaf width and length, plant size, volume and development on plant and organ level. The introduced traits were shown with respect to the measured plant types, the used measuring technique and the link to their biological use case. These were trait and growth analysis for measurements over time as well as more complex investigation on water budget, drought responses and QTL (quantitative trait loci) analysis. The used processing pipelines were generalized in a 3D point cloud processing workflow showing the single processing steps to derive plant parameters on plant level, on organ level using machine learning or over time using time series measurements. Finally the next step in plant sensing, the fusion of different sensor types namely 3D and spectral measurements is introduced by an example on sugar beet. This multi-dimensional plant model is the key to model the influence of geometry on radiometric measurements and to correct it. This publication depicts the state of the art for 3D measuring of plant traits as they were used in plant phenotyping regarding how the data is acquired, how this data is processed and what kind of traits is measured at the single plant, the miniplot, the experimental field and the open field scale. Future research will focus on highly resolved point clouds on the experimental and field scale as well as on the automated trait extraction of organ traits to track organ development at these scales.

## Background

Measuring three-dimensional (3D) surface information from plants has been introduced during the last three decades [1–3]. Having access to the plant architecture [4] enables tracking the geometrical development of the plant and the parameterization of plant canopies, single plants and plant organs. As 3D measuring is non-destructive the implementation of a monitoring over time is possible [5]. Doing this in 3D is essential to differentiate between plant movement and real growth on plant and organ level [6]. Plant phenotyping defines the goal of bridging the gap between genomics, plant function and agricultural traits [7]. Therefore 3D measuring devices are a well-suited tool as these devices enable exact geometry and growth measurements.

This can be reached using different techniques as there are laserscanning, structure from motion, terrestrial laser scanning or structured light approaches, as well as time of flight sensors or light field cameras. Each of these technologies has its own use cases for (single) plant scale (laboratory, < 10 plants), miniplot scale (greenhouse, < 1000 plants), experimental field (< 10,000 of plants) or use on an open field (< 10,000 of plants) to meet the different requirements regarding robustness, accuracy, resolution and speed for the demands of plant phenotyping as there are the generation of functional structural plant models to link the geometry with function, [8] to differentiate between movement and growth to visualize and measure diurnal patterns [6] to monitor the influence of environmental stress to the plant development [9].

All techniques result in a point cloud, where each single point provides a set of X, Y, Z coordinates that locate the point in the 3D space. Depending on the measuring device, this coordinate can be enriched with intensity- or color-information representing the reflected light into the direction of recording. Existing 2.5D approaches measure distances from one single point of view. In contrast to this real 3D models depict point clouds recorded from different views showing different spatial levels of points and thus show a smaller amount of occlusion, a higher spatial resolution and accuracy. Furtheron resolution is defined as the smallest possible point to point distance for a scan—also known as sampling distance. Accuracy depicts the distance between real and measured target point.

**Fig. 1:**
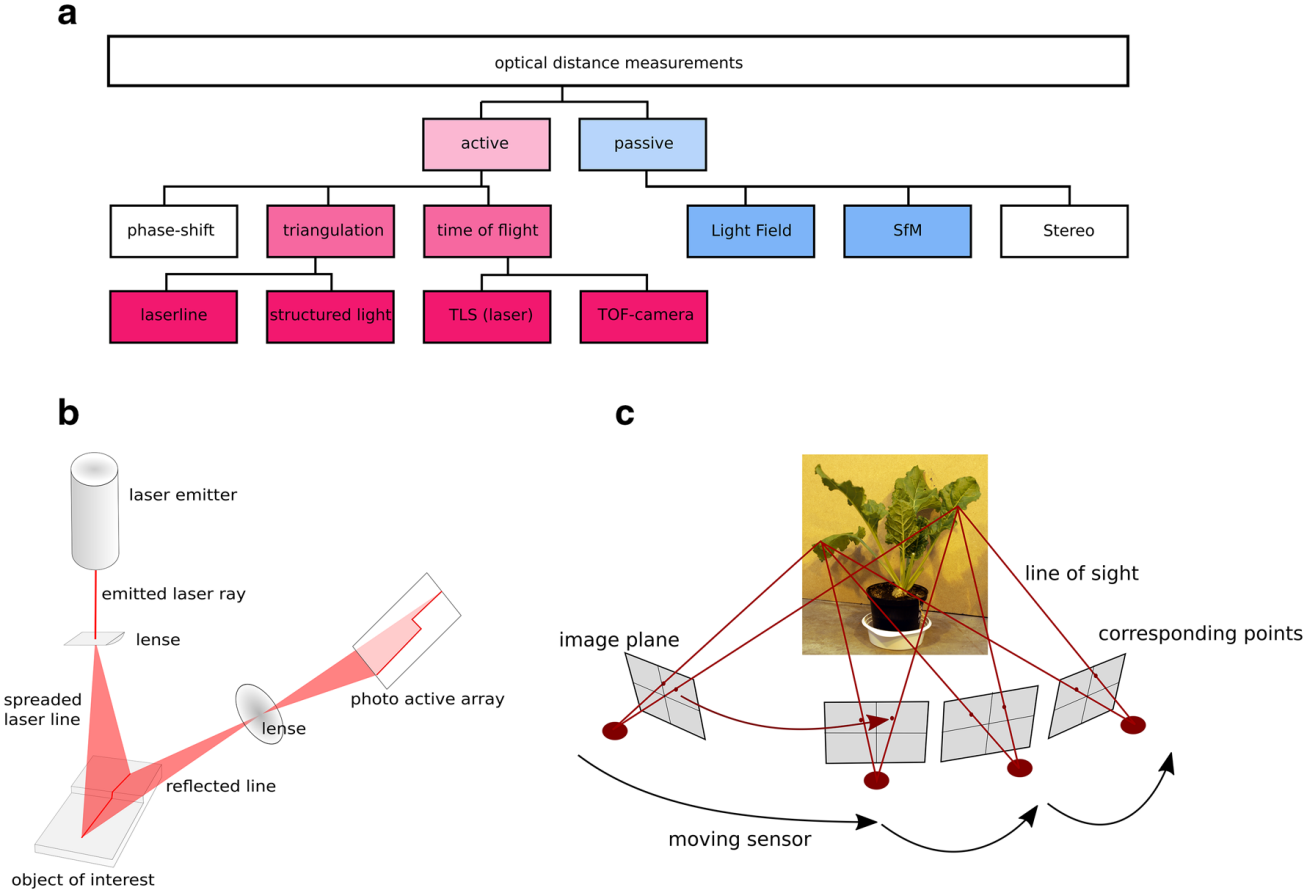
The hierarchy of the introduced 3D measuring techniques which are most relevant for plant phenotyping (highlighted in color) is presented. Laser triangulation, structured light approaches, time of flight sensing, structure from motion and light field imaging are shown in their technical connection (**a**). The two most important techniques laser triangulation (**b**) and structure from motion (**c**) are introduced in detail to show the procedure of point measuring

A technical categorization of 3D measuring techniques is shown in Fig. 1a. It mentions the two main categories which use active illumination based and passive approaches. Active illumination describes sensors that use an active light emitter, passive sensors use the environmental light condition to measure. Triangulation based systems and time of flight measurements are active measurement techniques. Triangulation based techniques are laser triangulation (LT) and structured light (SL) techniques, time of flight based techniques are terrestrial laser scanning (TLS) and time of flight (ToF) cameras. Light field cameras (LF) and structure from motion (SfM) approaches belong to the group of passive methods. A more technical description with focus on the output and price is shown in Table 1.

This review aims to giving an answer to significant questions regarding 3D plant phenotyping. What are the point cloud requirements used for 3D plant phenotyping at different scales regarding point resolution and accuracy? What are the sensor techniques that can be used for specific plant phenotyping tasks? How are these datasets processed, what kind of traits have been extracted and what is their biological relevance?

### Laser triangulation, LT

LT is mostly applied in laboratory environment due to its high resolution and high accuracy measurements [10] or due to its easy setup using low-cost components [11, 12]. Laserscanning describes systems based on laser distance measurement and a sensor movement. Typically this means the use of a laser triangulation system. Hereby a laser ray is spread into a laser line to illuminate the surface of interest. The reflection of the laser line is recorded using a sensitive photoactive array (CCD or PSD). The calibration of the setup enables an interpretation of the measurement on the camerachip as a distance measurement (see Fig. 1b). A complete 3D point cloud can be extracted by moving the sensor setup. LT systems work with active illumination and can be used independently of the outer illumination. A point resolution of a few microns can be reached [10].

LT setups always include a trade-off between possible point resolution and measurable volume. Either a small volume can be measured with highest resolution or a big volume is measured in low resolution. This requires a sensor system adaption for a complete experiment before and a good estimation of the necessary resolution and measurable volume.

Adapted sensor systems aiming at plant point clouds with a resolution of millimeters have risen within the last few years. These sensors use laser triangulation for measurements on field scale using non-visible laser wavelength (NIR, usually 700–800 nm), which results in a better reflection under sunlight [13, 14].

### Structure from motion, SfM

SfM approaches use a set of 2D images captured by RGB cameras to reconstruct a 3D model from the object of interest [15]. After estimation of intrinsic (distortion, focal length etc.) and extrinsic (position and orientation) camera parameters the images were set into context [16] using corresponding points within the images (see Fig. 1c). These corresponding points are used to connect the images and to calculate the 3D model. Depending on the camera type the result is a 3D point cloud including color (RGB) or intensity of the measured reflection [17]. The resolution is comparable to LT point clouds but it strongly depends on the number of images used for 3D calculation, the amount of different viewing angles from where the pictures were taken as well as from the camera chip (CCD) resolution [17].

In contrast to LT where most effort is needed during measuring and the immediate result is the point cloud, SfM approaches need a short time for capturing the image, but need much effort for the reconstruction algorithm.

SfM approaches are mostly used on UAV (unmanned areal vehicle) platforms as they do not need a special active illumination or complex camera setups. As this approach just needs a camera for the image acquisition the hardware setup is very small and lightweight. Thus, this approach fulfills the lightweight demands that were defined by UAV restrictions on weight. As cheap consumer cameras can be used and the algorithms are mostly free to use, this technique is commonly used for modelling input models for 3D printers from the non-professional community using handheld or tripod mountings. Thus many applications are available focusing not on accuracy but reproducibility.

### Structured light (SL) and time of flight (ToF) and light field (LF) and terrestrial laser scanning (TLS)

There are various other techniques to image three-dimensional data beside LT and SfM approaches. Most common are SL, ToF and LF approaches. SL uses patterns, mostly a grid or horizontal bars, in a specific temporal order. For each pattern an image is recorded from the camera. By using a pre-defined camera-projector setup the 2D points on the pattern are connected to their 3D information by measuring the deformation of the pattern [18, 19]. As SL setups are rather big regarding the used space for the measuring setup and need a lot of time to acquire the images either the object or the measuring system has to be moved to connect different points of view. SL approaches are implemented in industry to perform reverse engineering or for quality control providing high resolution and high accuracy in a bigger measuring volume [20].

ToF uses active illumination, the time between emitting light and returning of the reflection is measured by using highly accurate time measuring methods [21]. This can be performed for thousands of points at the same time. ToF cameras are small regarding the hardware size but capture images with a rather small resolution. These cameras are mostly used for indoor navigation [22] or in the gaming industry (see Kinect 2, [23]).

LF cameras [24] provide, beside a RGB image, additional depth information by measuring the direction of the incoming light using small lenses on each pixel of the camera array. This enables reconstruction of 3D information.

Tof and LF Setups have to be moved to get a complete 3D point cloud, but as ToF is rather slow it suffers on a low resolution similar to LF approaches, when compared to LT and SfM measuring approaches (see Fig. 1).

A technique coming from land surveying is terrestrial laser scanning. Using a time of flight or a phase shift approach these scanners scan the environment and have to be moved to another position to capture occlusions. Nevertheless these systems are very well established for surveying jobs like landslides detection of deformation monitoring of huge areas [25]. For plant monitoring their advantage of big measurable volume ($< 300$ m), accuracies of millimeters are possible but surveying knowledge is needed especially when using more than one point of view. Nevertheless the technique is well established tool for canopy parameters. Nevertheless as it is cost intensive, hard to process as the different position measurements have to be connected and its time consuming measuring procedure it is not very appropriate for plant measuring.

**Table 1:** Technical overview of 3D measuring methods

Name	Abbr.	Resolution	Active illumination	Direct point cloud access	Output values	Price	Literature
Laser triangulation	LT	< mm	Yes	Yes	XYZ (I)	€€€	[10, 26]
Structured light approaches	SL	< mm	Yes	No	XYZI (RGB)	€€	[18, 27]
Structure from motion	SfM	mm	No	No	XYZRGB	€	[17, 28]
Time of flight	ToF	mm	Yes	Yes	XYZ (I)	€€	[29, 30]
Light field measuring	LF	mm	No	No	XYZRGB	€€€	[31, 32]
Terrestrial laserscanning	TLS	cm	Yes	Yes	XYZ (I/RGB)	€€€	[33, 34]

The differences in illumination, direct or needed postprocessing, pricing and resulting values is given. Pricing is encoded by € < 1000 Euro, €€ < 10,000 Euro and €€€ > 10,000 Euro. The output values show *XYZ* coordinates and a possible enrichment with *I* for intensity/reflectance or *RGB* for a Red-Green-Blue image combination

## Point cloud resolution—its effect on the extracted traits

To answer the question for the needed requirements on point clouds and thus on 3D measuring devices for the demands of plant phenotyping it is important to compare these tools regarding their accuracy with established tools for trait measuring. 3D plant measuring has proven to be a reliable tool for plant phenotyping when compared to established manual or invasive measurements [3].

Nevertheless the comparison between proven non-invasive technologies as well as the requirements regarding the scan resolution for an accurate measurement in a specific scenario remains an open question.

An experiment was conducted to show the comparison between a high precision LT system and a non-invase established technology—a leafmeter. Both techniques were compared to an established, but invasive photo based reference method [35, 36] with an accuracy of below mm. The photo based method uses a RGB image and a metric reference frame and comes together with destruction of the plant as the leaves were cutted and positioned within the metric frame. The reference experiment includes ten different barley plants. Each plant had six to seven leaves, where at least five leaves have been measured due to constraints of the leafmeter which makes it impossible to measure the inner leaves. During the measurement the plants were in the BBCH 30 growth stage. The plants were cultivated in a greenhouse. For the measurements a leafmeter (Portable Laser Leaf Meter CI-203, CID Inc., Camas, WA, USA) was used as a well established tool for leaf area measuring [37] and a laserscanner (Romer measuring arm + Perceptron v5, [38]) were used. The laser scanner point cloud consists of several thousand 3D lines, which were automatically merged. To receive an evenly distributed point cloud it has been rastered (0.3 mm point to point distance) and meshed using a surface smoothing approach as it has been provided by CloudCompare (version 2.10 Alpha, http://www.cloudcompare.org). The leaf area was calculated by summing up the area of all triangles of the mesh, a method that has been applied to corn measurements before [39]. The error metric (RMSE and MAPE) was calculated according to [3].

**Fig. 2:**
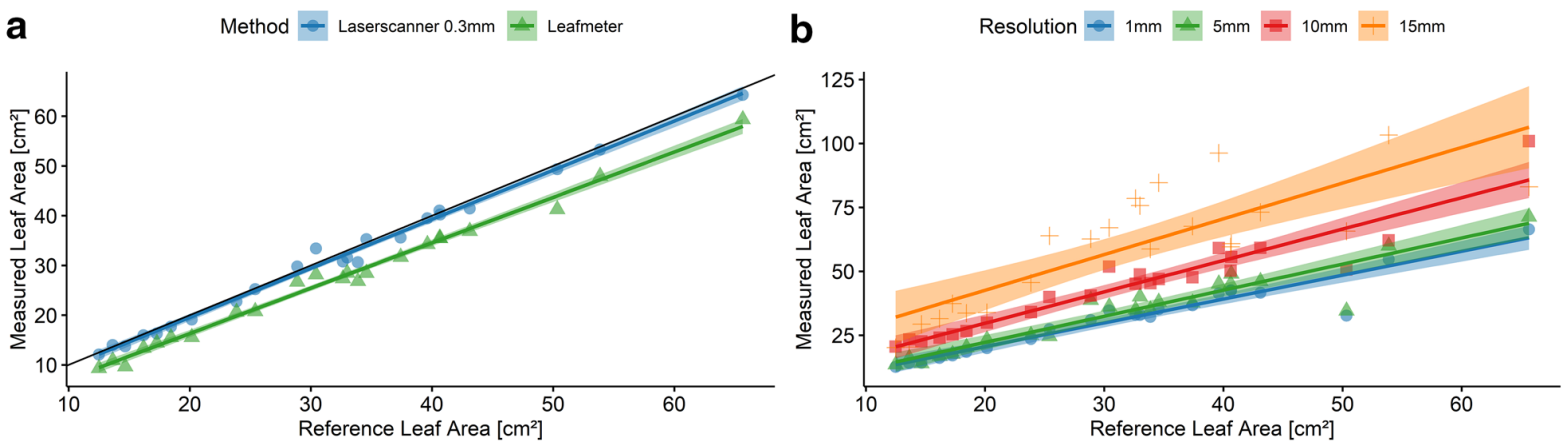
Laserscanning accuracy—reference experiment using a photogrammetric method as reference to evaluate the accuracy of the Laserscanning device and the Leafmeter as a device for measuring leaf area [35, 36]. Both methods show a high correlation compared to the reference method (**a**). The comparison between the laser scanner using different point resolutions and the introduced reference method is visualized in addition (**b**). The transparent color in both plots indicates the confidence intervals (95%). The black line describes the bisecting line of the angle as the line of highest correlation

Figure 2a shows a correlation between laserscanner and reference measurements ($R^2=0.99$), same for the leafmeter and the reference measurements ($R^2=0.99$). The leafmeter shows a small offset due to its way of handling, as there is a small offset while positioning the leafmeter at the leaf base. Error measurements were provided in Table 2.

By reducing the laser scanned point cloud regarding resolution and point accuracy the error levels compared to the established leafmeter can be determined.

A further analysis focusing on the applicability of different point resolutions (1–15 mm) was conducted as the introduced 3D measuring techniques provide differences regarding resolution and accuracy (Fig. 2b). Therefore, the scans of the first experiment were resampled and the amount of points was reduced. In addition, noise in the dimension of the resolution (1–15 mm) was added to the single points to simulate other 3D sensing sensors and technology in a more accurate way. In Table 2 the results of the correlation analysis and related error measurements were described. Errors were below $1\%$ (MAPE) for all point clouds with reduced quality compared to the reference measurement. Point resolutions above 15 mm were not investigated as not enough points were left to model the leaf.

As expected with decreasing resolution the error is increasing. A laser based 3D measuring device that provides a resolution of 5 mm is comparable with a leafmeter regarding the proportional error measurement. Down to a resolution of 15 mm the percentage error was still below $1\%$ although the RMSE raised up to 30 cm^2^. This means, that even with low resolution 3D measuring devices exact trait measurements are possible.

**Table 2:** Comparison of the error measurements for the leafmeter–laserscanner combination using different sampling resolutions for the laserscanned point cloud between 0.3 and 15 mm

	Resolution [mm]	$R^2$	MAPE %	RMSE cm^2^
Leafmeter		0.99	0.16	5.01
Laserscanner	0.3	0.99	0.04	1.34
	1.0	0.91	0.05	3.94
	5.0	0.89	0.11	5.4
	10.0	0.89	0.43	13.96
	15.0	0.68	0.90	30.66

Sampling included a reduction in resolution as well as adding noise in the same dimension. $R^2$ error measurements are shown as well as a MAPE and RMSE calculation

**Fig. 3:**
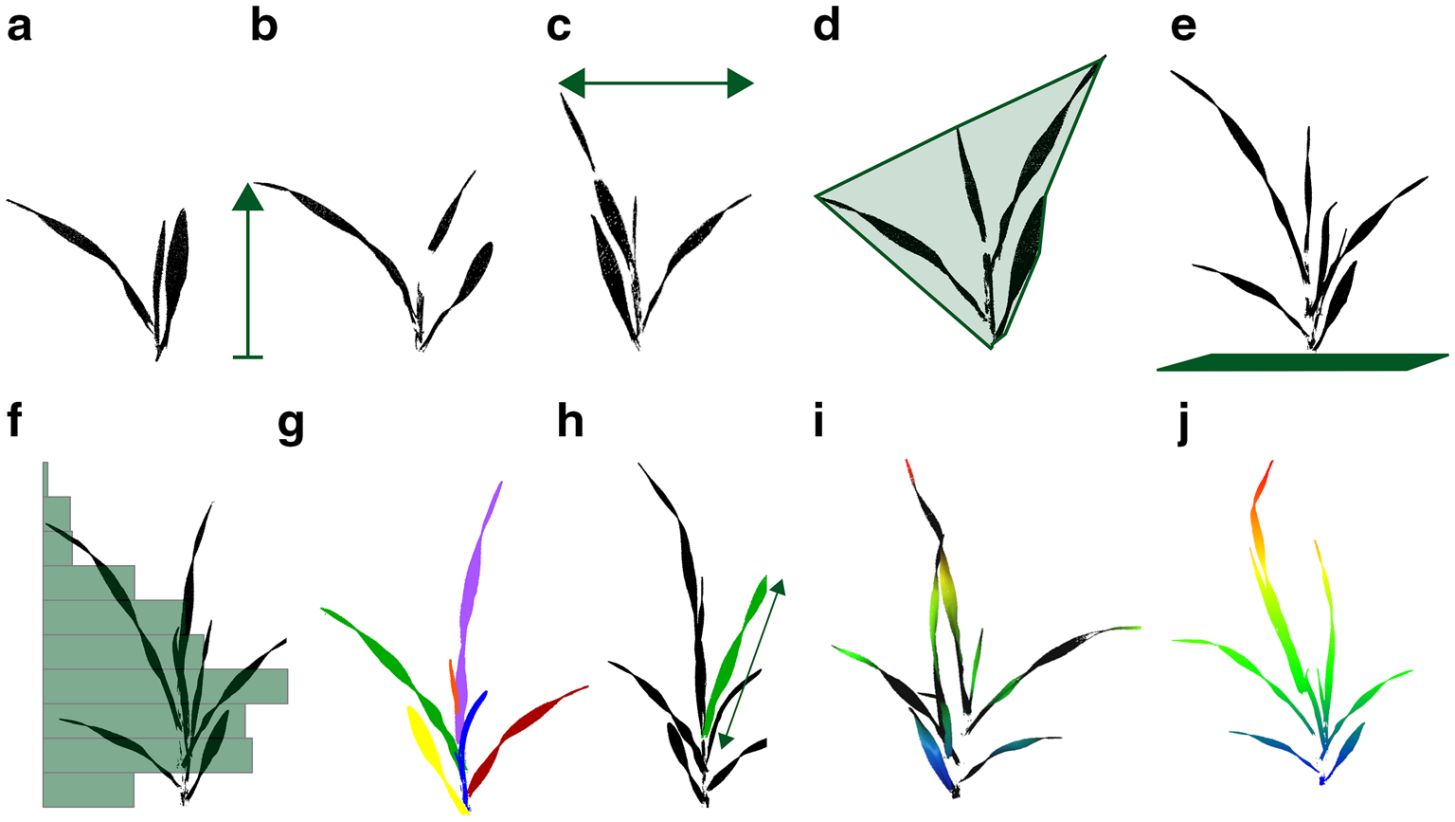
Traits that can be extracted from a 3D point cloud of a young barley plant. From the XYZ point cloud (**a**) non-complex parameters like plant height (**b**), plant width (**c**), the convex hull (**d**) and the projected leaf area (**e**) can be extracted. Furthermore the leaf area density (**f**) can be derived. The number of leaves (**g**) and the respective leaf length (**h**) can be measured after identifying the individual plant organs. For each point the inclination and its height can be calculated resulting in a inclination (**i**) or height map (**j**)

## Data processing and 3D trait analysis

3D scanning of plants enables capture of the geometry of the plant and individual organs like leaves or stems. Thus, parameters for the whole plant and the organs can be calculated to describe size, shape and development. Subsequent traits using the complete plant point cloud (canopy) are depicted as non-complex traits, whereas parameters describing geometry at the organ level are depicted as complex traits, as they require a previous identification of plant organs by using classification routines. Non-complex traits are height, width, volumetric measures, maps showing information about height or inclination or a rough leaf area estimation. The latter describes the trait leaf area from a non-segmented point cloud where a large percentage of the points are leaf points. Complex plant traits describe plant traits on organ level such as the exact leaf area, stem length, internode distance, fruit counting or ear volume estimation. By repeating these measuring/analysis setups over time the extraction of time lapse traits like leaf surface development, leaf movement or field maps showing the growth at different locations is possible. As time can be described as an additional dimension time lapse traits are named 4D traits.

Even non-complex traits often need a definition before a comparison to well-established measuring tools is possible. For example the internode distance can be depicted to be the distance between two consecutive leaf petiole at the stem, or as the distance between leaf centre points projected to the plant stem [27].

Figure 3 illustrates the derivation of traits from a barley point cloud without any reflection information. It shows the derivation of the parameters plant height, plant width, convex hull, projected leaf area, the leaf area density, the number of leaves, the single leaf length as well as height and inclination maps.

Height or width can be extracted by using the difference between lowest and highest z-axis coordinate for height and same for x- and y-axis to get a measurement for the width [3]. A more complex trait is the convex hull. In 2D this describes the smallest convex polygon covering all the points. It approximates the volume of the plants in 3D [3]. The projected leaf area represents the cover of the ground by the plants leaves. It is widely used to characterize canopy light conditions and is used to calculate (projected) leaf area index [40]. The height distribution of leaf surface points it is an indication for variation in leaf mass per area as it was shown for rice between different varieties and different nitrogen levels [41]. The number of leaves is one important trait as it is used, among others, to describe the growth stage of plants in the BBCH scale [42]. Unfortunately accessing the leaf number automatically is difficult. For 2D plant images this problem has already been addressed but it was noted to be rather complicated [43]. Existing datasets have been used to raise a challenge to solve this problem [44]. In 3D different methods can be used to identify the plant organs and to give semantic meaning to the point cloud or respectively to the organs. There are approaches using meshing algorithms [45] that uses the mesh structure for segmentation, approaches that fit the plant measurements into a model [46], others use the point environment within the point cloud and machine learning methods like Support Vector Machines coupled to Conditional Random Field techniques to overcome errors in the classification to identify the organs [47]. Further methods are Region Growing [48] and clustering routines [49] and Skeleton Extraction approaches [50] which can be used. Nevertheless, results of these approaches correlate with the quality of the underlying point cloud.

When the single leaves are identified the parameterization can be performed on organ level to calculate the leaf area of single leaves. Paulus et al. [3] showed an approach for manual leaf tracking and to monitor the leaf development over time. Leaf organs can be parameterized by using a triangle mesh. Here the sum of all triangles corresponds to the leaf area. Organs like the plant stems need a more sophisticated parameterization. Mathematical primitives like cylinders show a good approximation of the stem shape [51] and enable extract measurements like height or volume [5]. Further analysis of point height distribution mostly is used to generate maps to identify areas of differences in growth [13].

**Fig. 4:**
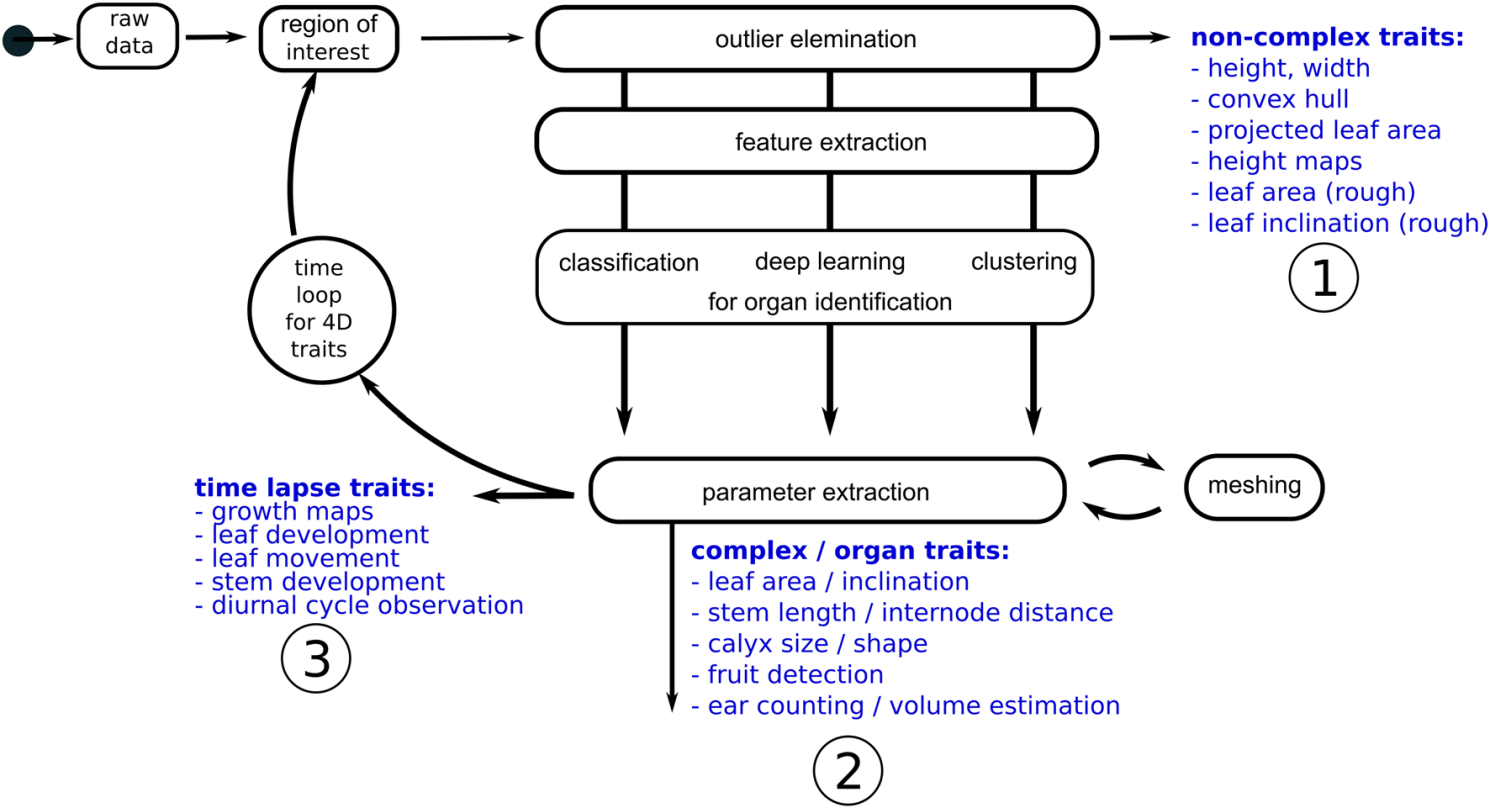
A common 3D processing pipeline including the use of a region of interest and outlier handling to extract non-complex parameters as height, width and volume (1). The use of routines like machine learning/deep learning enables the identification and parameterization of plant organ parameters (2). Using multiple recordings over time monitoring of development and differentiation between growth and movement is possible (3)

Figure 4 shows a processing pipeline for 3D point clouds coming from a common point cloud generating 3D scanning device. After cutting the point cloud to the region of interest and a first cleaning step using an outlier removal algorithm non-complex parameters like height and width can be derived based on the point cloud parameters. Using routines from standard data processing software libraries like Matlab (MATLAB, The MathWorks, Inc., Natick, Massachusetts, United States.), OpenCV [52] or the Point Cloud Library [53] non-complex traits like the convex hull volume, projected leaf area or height maps can be extracted. By use of plane fitting and meshing algorithms parameters, leaf area and inclination can be calculated (see Fig. 4, part 1).

Further processing uses either machine learning approaches to identify (segment) plant organs like leaf, stem or ears [54]. These routines work on 3D features like surface feature histograms or point feature histograms [55, 56] and encode the surface structure. Machine learning algorithms such as Support Vector Machines (as provided by LibSVM [57]) needs pre-labeled data for training and belong to the supervised learning methods. They can be applied if labeled data is available and use this training data to develop a model for classification. Unlike this, methods that use the structure within the data are called unsupervised learning methods, they do not need any labelling but they are hard to optimize. 3D geometry features and clustering methods have been successfully applied to divide barley point clouds into logical groups for stem and leaf points [49] (see Fig. 4, part 2).

Measurements over time (4D) and repeated application of the described workflow growth parameters for plant development like growth curves on plant and organ level can be derived. As 3D devices enable a differentiation between growth and movement the diurnal cycle can be observed and can be compared to the daily growth [6]. As growth is a direct indicator of stress high precision 3D measuring devices are well suited to detect this stress by measuring the 3D shape change [2] (see Fig. 4, part 3).

## 3D parameters on different scales

The following section gives an overview of different parameters that have been described in literature. The parameters have been grouped into four different scales “Single Plant”, “Miniplot”, “Experimental Field” and “Open Field”. Single plant scale as it is focused in laboratories describes the scale from seedlings to fully grown plants but with a focus on single plants or smallest groups of plants. Here, high resolution sensors (< mm) working in a reproducible setup with highest accuracy were used. Miniplots in greenhouses describe production farms with fixed plant locations as well as high throughput plant phenotyping facilities where the plants stand on conveyor belts and were imaged in imaging cabinets. These setups are commonly used for research studies [58]. The experimental field scale describes measurements in the field with stationary sensors, maybe on a tripod or slowly moving sensor platforms. The largest scale shown here describes open fields. Sensors that are used here are commonly mounted on UAV platforms. These sensors provide a lower resolution (cm), but a high scan speed (> 50 Hz), which is essential when used during motion. The accuracy measurements (see Table 3) are based on a linear correlation using $R^2$ notation or the use of the MAPE [3].

**Table 3:** Overview of plant traits that have been measured for the single plant scale, miniplot, experimental field and open field scale

	Trait	Plant	Sensor	Biological connection	Literature
Single plant scale	Plant height	Sugar beet	LT	Drought response	[3]
	Plant width	Sugar beet	LT	Drought response	[3]
	Root volume	Sugar beet	LT	Trait analysis	[11]
	Root surface	Sugar beet	LT	Trait analysis	[11]
	Root compactness	Sugar beet	LT	Trait analysis	[11]
	Leaf area	Barley	LT	Drought response	[3]
	Projected leaf area	Sugar beet	LT	Trait analysis	[11]
	Leaf width	Cotton	SfM	Growth analysis	[45]
	Leaf length	Cotton	SfM	Growth analysis	[45]
	Leaf movement	Arabidopsis	LT	Growth analysis	[6]
	Single leaf growth	Barley	LT	Growth analysis	[3]
	Number of leaves	Cabbage	SL	Trait analysis	[27]
		Cucumber	SL	Trait analysis	[27]
		Tomato	SL	Trait analysis	[27]
	Stem length/growth	Barley	LT	Growth analysis	[5]
	Calyx shape	Strawberry	SfM	Trait analysis	[59]
	Achene shape	Strawberry	SfM	Trait analysis	[59]
	Internode distance	Cabbage	SL	Trait analysis	[27]
		Cucumber	SL	Trait analysis	[27]
		Tomato	SL	Trait analysis	[27]
	Ear volume	Wheat	LT	Yield estimation	[54]
	Ear shape	Wheat	LT	Yield estimation	[54]
Miniplot	Plant height	Pepper	SfM	QTL analysis	[60]
	Leaf angle	Pepper	SfM	QTL analysis	[60]
	Leaf area	Rapeseed	LT	Growth analysis	[14]
	Proj. leaf area	Rapeseed	LT	Growth analysis	[14]
	Leaf angle	Maize	ToF	Trait analysis	[24]
		Sorghum	ToF	Trait analysis	[24]
		Soybean	SFM	Drought response	[61]
	Fruit detection	Tomato	LF	Trait analysis	[21]
Experimental field	Plant height/canopy height	Wheat	LT	Growth analysis	[13]
	Proj. canopy area	Cotton	TLS	Growth and yield	[62]
	Plant volume	Cotton	TLS	Growth and yield	[62]
	Leaf area index (LAI)	Maize, sorghum	SfM	Trait analysis	[63]
	Leaf area	Grapevine	SfM	Trait analysis	[64]
		Peanut	LT	Water budget	[65]
		Cowpea	LT	Water budget	[65]
		Pearl millet	LT	Water budget	[65]
Open field	Plant height and canopy height	Maize	SfM	Growth analysis	[66]
		Sorghum	SfM	Growth analysis	[66]
		Eggplant	SfM	Biomass estimation	[67]
		Tomato	SfM	Biomass estimation	[67]
		Cabbage	SfM	Biomass estimation	[67]

If possible an error measurement is provided as well as the plant type, the sensor and the biological connection as the purpose of the study

To define the different scenarios of applications on the plant, miniplot, experimental field and open field scale. Table 3 provides an overview of measured plants, traits and biological connection.

Multiple studies focus on scenarios with just a few plants in laboratories. Here a differentiation between single organs is mostly not necessary. Non-complex parameters that are easy to measure like height, volume, number of leaves or projected leaf area have been extracted with high precision ($R^2>0.9$, [17]). A further step that needs either a modelling of the plant [45] or the use of a sophisticated classifier working on the pure point cloud [54] enables a differentiation between the single organs. This can be used for wheat ear volume calculation for yield estimation [54] using the $\alpha$-shape technique or measuring of stem parameters by using cylinder fitting routines [3].

On the miniplot scale, which comes along with similar prerequisites regarding resolution and accuracy like the single plant scale, there are further demands regarding recording speed as it is essential for high throughput phenotyping using automated greenhouses and conveyor systems. For trait and growth analysis laser triangulation systems [14] are very common, but time of flight sensing [24] and structure from motion [64] approaches are also used, mostly due their high speed during the recording, although a not negligible amount of processing time is needed after the scan pass. In comparison to the single plant scale parameters assessed here commonly are non-complex parameters like height or leaf area where the stem points were neglected due to the smaller resolution or lower proportion of measured points.

Experimental field measurements concentrate on parameters like plant/canopy height [13], volume [62] or leaf area index [63]. At this scale terrestrial laser scanners are often used as they provide a range of 10s to 100s of meters and a high resolution of a few millimeters [62]. Structure-from-motion approaches are used on wheeled carrier vehicles with mounted cameras [64] as well as on UAV-based measurements. The latter comes along with measurements of easy accessable parameters like plant height or canopy volume and can be utilized for growth analysis and biomass estimation [66, 67].

Table 3 introduces the biological connection of the 3D parameters as there are links to trait analysis, growth analysis, drought responses, analysis of water budget, yield estimation, biomass estimation and QTL analysis (quantitative trait loci, [68]).

By comparing different groups of plants regarding their responses on water access drought can be described [3, 61]. Combining 3D measurements with gravimetric measurements of the transpiration enables measuring the water budget and the transpiration rate over day on a single plant scale [65]. These experiments use a non-destructive measuring method to link an accompanying sensor to 3D plant traits. Using destructive yield measurements enables linking the 3D traits to yield parameters like thousand kernel weight or kernel number as shown for wheat [54]. Similar to this, the scan of a complete plant can be linked to fresh mass/biomass even on field scale [67]. QTL analysis describes the identification of genetic regions that are responsible for specific plant traits. 3D measuring helps to identify and describe traits that are linked to these regions [60] and to understand the genotype-phenotype interaction.

### Adding information to the 3D data

The phenotype as the result of genotype and environment interaction is expressed in numerous plants traits which are not all expressed in geometrical differences. Therefore different sensors were taken into account. RGB cameras are common in plant phenotyping being used to extract different traits regarding size, shape and colour [69]. Multispectral- or hyperspectral cameras are used to identify indications or proxies in the non-visible spectrum to detect plant stress [70] or plant diseases [71]. Thermal cameras show differences in temperature between plants or within a single plant [72].

**Fig. 5:**
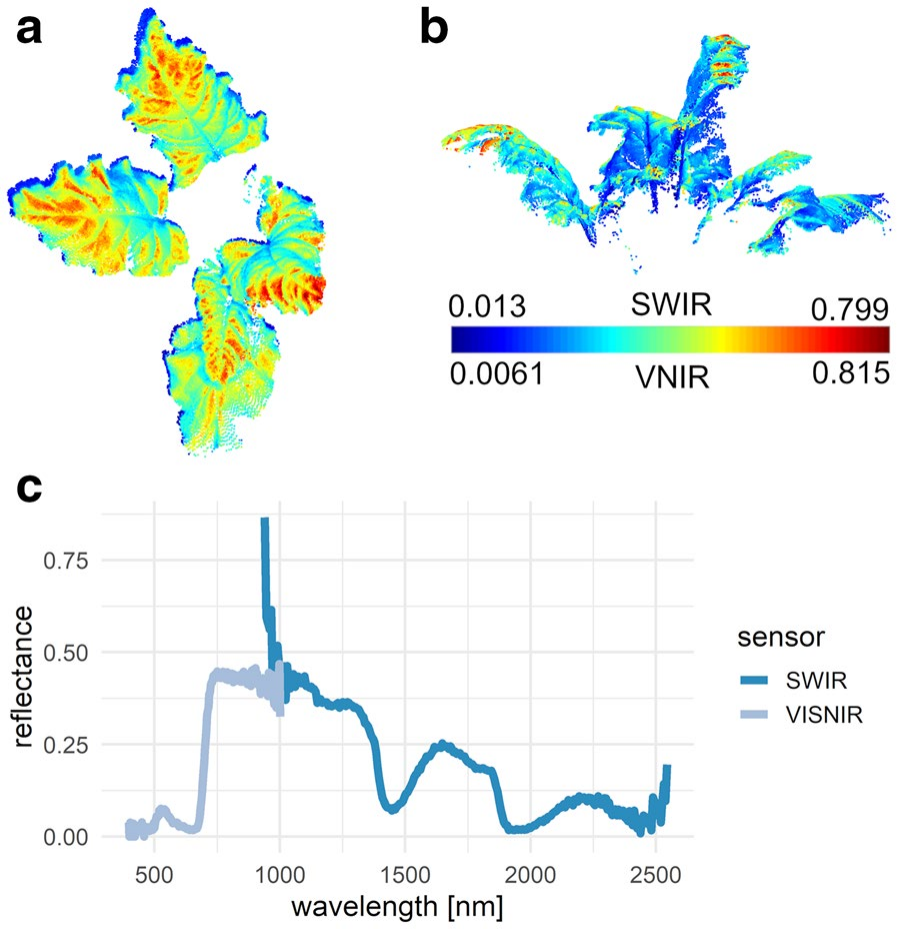
A combination of 3D point cloud and hyperspectral image data is possible by calibrating the sensor setup including the 3D imaging sensor and the hyperspectral camera. The top (**a**) and side (**b**) view of a combined point cloud is shown for combination of 3D- and VISNIR-data (911 nm, **a**) as well as for 3D and SWIR data(1509 nm, **b**). The VISNIR and the SWIR spectrum can be investigated at the same point in the 3D point cloud (**c**)

Depending on the plant surface geometry these recording devices vary in their measurements. [73] showed a connection between high NDVI (normalized difference vegetation index [74]) values and the inclination angle on sugar beet leaves. By using the plant’s 3D information the effect of different reflection angles with respect to illumination source, camera and observed surface can be recorded [75]. For combining 3D and hyperspectral images the camera system has to be geometrically modelled. The result is a combined 3D-reflection model that combines 3D geometry and reflection information from hyperspectral cameras (see Fig. 5). As it is advantageous to take this into account and to reduce the described error future measurement hardware should include this correction method internally as by a proper modelling of the optical ray path [76].

## A critical consideration on 3D scanning of plants

The shown experiment (see Fig. 2) and the the intense literature work (see Table 3) indicate that 3D measuring devices and especially laserscanning devices are reliable tools for plant parameterization with respect to plant phenotyping. Existing invasive tools can be replaced and exceeded in accuracy. Furthermore an estimation for the required resolution for a laboratory/greenhouse experiment was given together with resulting error measurements. A MAPE of 5–10% was previously defined to be acceptable for morphological scale phenotyping, as this limit reflects the magnitude of errors already inherent in manual measurements and which is low enough to to distinguish changes in relevant traits between to imaging dates during development [45]. Although the resolution and point accuracy was decreased down to 15.0 mm within this experiment the MAPE measurement never broke this limit.

One big advantage that is shared by all 3D measuring devices is the fact that the point cloud represents the surface at a specific point in time. As this is, a very general representation of the plant surface and not a single measurement, many different traits can be extracted even afterwards. If leaf area is focused in an experiment and later on leaf inclination becomes relevant, this trait can be calculated afterwards and compared to the current experiment [77].

All the 3D measuring methods have in common that with increasing age of the plants the complexity and thus the amount of occlusion is increasing. This can be reduced by using more viewpoints for each sensor. Nevertheless occlusion is always present independent of sensor, number of viewpoints or sensor setup as the inner center of the plant is at a specific time occluded by the plant (leaves) itself. One solution could be to use MRI (magnetic resonance imaging) or radar systems that use volumetric measurements [78], taking into account a more complex and expensive measuring setup. Depending on the measuring technique the registration (fusion) of different views is rather difficult when wind occurs or plants were rotated during single scans. Referencing becomes impossible and the results loses quality. This holds for almost any 3D measuring technique as long as imaging is not performed in one shot from many different positions at the same time as it has been already published for tracking of human motion [79].

Although 3D measuring devices provide a very high resolution they are only able to measure visible objects. Plant roots can be imaged when growing in transparent soil like agar. Their traits can be distinguished into static and dynamic root traits, depending if they can be measured at a single point in time (static) or at multiple points in time (dynamic) [80]. The latter can be related to growth and spatiotemporal changes in root characteristics, but only the static traits can be measured by 3D devices as the roots have to be taken from the soil, washed and measured. One effect that has to be taken into account is the problem of refraction when measuring through different substances.

In general, LT is able to cover applications where a high resolution and accuracy is needed in a rather small measuring volume as it is essential for organ-specific trait monitoring on the single plant scale. Whereas Sfm covers most of the application scenarios in plant phenotyping across all scales as the resolution and the measuring volume just depend on the camera and the amount of acquired images. The more data from different points of view is merged independent of the sensor the less occlusion can be found in the resulting point cloud.

### Summing up LT

To resolve smallest details the high resolution of microns using LT technique is well prepared. Its exact point clouds are a well suited input for machine learning methods to extract parameters of plant organs like stem length or calyx shape. Nevertheless, the interaction between laser and plant tissue has to be taken into account when using measuring systems with active illumination and laser triangulation in special. Although laser scanning is depicted to be non penetrating, latest experiments have shown that plant material below the cuticula and lasercolor and intensity have a significant influence on the measuring result and its accuracy [81, 82]. Furthermore the edge effect, measurements of partly leaf and partly background, can lead to outliers or completely wrong measurements [83].

### Summing up SfM

SfM approaches provide a quick acquisition and are lightweight. This makes them well suited for use on flying platforms to image field trials. The more images recorded the better is the resolution of the resulting point cloud. SfM approaches provide a high accuracy (mm) [17], but strongly interfere with illumination from the environment. Light is problematic when it is changing during or between consecutive measurements. Furthermore wind is a problem as the object moves between two consecutive recordings. This causes errors during the reconstruction process [84]. This can be reduced by using a high measuring repetition rate (> 50 Hz) but this raises the time needed for reconstruction (> 1 h). Latest research focuses on reducing the post-processing time [85] as it is a key capability for autonomous driving. As autonomous driving is strongly pushed forward, a huge increase regarding the performance of the reconstruction algorithms is be expected.

### Summing up SL, ToF, TLS and LF

SL, ToF, TLS and LF measurements have shown their applicability for the demands of plant phenotyping. Nevertheless the accuracy and resolution have to be increased for the demands of high throughput plant phenotyping. There are prototype setups where these techniques are the method of choice.

### Further methods

In addition to the shown devices for 3D imaging of plants on the different scales there are more devices like 3D measuring systems for the microscopic scale using interferometry to localize the 3D position of proteins [86] or three-dimensional structured illumination microscopy to measure images of plasmodesmata in plant cells [87]. On a laboratory scale techniques like volume carving [88] were used for the determination of seed traits [89]. Magnetic resonance imaging (MRI) based techniques were used for 3D reconstruction of invisible structures [90] or in combination with positron emission tomography (PET) to allocate growth and carbon allocation in root systems [78]. Root imaging can also be performed using X-rays as a further technology that does not need visible contact to the object of interest to determine root length and angle [91]. On the beyond-UAV scale airborne methods were used like airborne laser scanning [2] to gather carbon stock information from 3D-tree scans. Measuring traits from trees has been done since many years [92]. Traits like diameters at breast height (DBHs) have been used to predict yield at trees [93, 94], but crops and vegetables grow much faster than forest trees.

### Opportunities and challenges

Visiting the introduced traits and methods the current challenges can be described as the transfer from the methods from the single plant scale to the field scale (experimental and open field). A requirement is the raising of the point cloud resolution which comes along with demands for sensor and carrier platforms. Sensors and algorithms have to overcome the limitations of the problems of plant movement (due to wind), the big amount of occlusion and the combination of different sensors together in a way that 3D information help to correct the influence of the geometry on radial measurements [73, 75]. Drones have to increase their accuracy as it could be provided by RTK GPS [95] or sensor fusion of on-board sensors for a better localization [96]. Nevertheless, 3D measuring sensors show a huge potential to measure, track and derive geometrical traits of plants at the different scales non-invasively. Further research should focus the definition of the traits, regarding the way plant height or internode distance is measured to enable a comparison of algorithms, plants and treatments among different research groups and countries.

## Concluding remarks

This review provides a general overview of 3D traits for plant phenotyping with respect to different 3D measuring techniques, the derived traits and biological use-cases. A general processing pipeline for use-cases in 3D was explained and connected to the derivation of non-complex traits for the complete plant as well as for more complex plant traits on organ level. If performing measurements over time the generation of growth curves for monitoring of organ development (4D) was introduced as well as their linking to biological scientific issues. Sensor techniques for the different scales from single plants to the field scale were recapped and discussed.

This review gives an overview about 3D measuring techniques used for plant phenotyping and introduces the extracted 3D traits so far for different plant types as well as the biological used-cases.

## Data Availability

The datasets during and/or analysed during the current study available from the corresponding author on reasonable request.

## Abbreviations

LT: laser triangulation
SfM: structure from motion
SL: structured light
LF: light field
ToF: time of flight
NIR: near infrared area (700–1000 nm)
SWIR: short wave infrared (1000–2500 nm)
UAV: unmanned areal vehicle
CCD: charged coupled device
PSD: position sensitive device
TLS: terrestrial laser scanning
QTL: quantitative trait loci
RSME: root mean square error
MAPE: mean absolute percentage error
BBCH: a plant development scale
